# Distribution difference of colostrum-derived B and T cells subsets in gilts and sows

**DOI:** 10.1371/journal.pone.0249366

**Published:** 2021-05-03

**Authors:** Ricardo Forner, Gabrielly Bombassaro, Franciana Volpato Bellaver, Shaiana Maciag, Francisco Noé Fonseca, Danielle Gava, Leticia Lopes, Mariana Groke Marques, Ana Paula Bastos

**Affiliations:** 1 Universidade Federal do Rio Grande do Sul, Porto Alegre, Brazil; 2 Instituto Federal Catarinense–Campus Concórdia, Concórdia, SC, Brazil; 3 Universidade Estadual do Centro-Oeste do Paraná- campus CEDETEG, Guarapuava, PR, Brazil; 4 Embrapa Suínos e Aves, Concórdia, SC, Brazil; INRA, FRANCE

## Abstract

Piglets are highly vulnerable to infections, but colostrum provides them with some protection. The function of colostrum components is unknown, as is if the amount and subsets of leukocytes in colostrum differ between gilts and sows. This study serially characterized leukocyte populations in colostrum for differential leukocyte counts. Differences in humoral and cellular composition of colostrum between 40 gilts and 40 sows (parities orders 3–4) from a commercial herd were examined. Flow cytometry is a useful tool to identify and quantify leukocyte subsets in sow colostrum. Overall, there were no (p ≥ 0.05) parity differences in total macrophages, granulocytes, and T and B cells. However, the sows’ colostrum presented significantly higher (p ≤ 0.05) T lymphocyte subsets than gilts, such as central memory CD4^+^T cells, effector memory CD4^+^T cells, and central memory CD8^+^T cells. Among B-lymphocytes, percentages of SWC7^+^CD5^+^ cells were significantly higher in sow colostrum than in that of gilts. As expected, IgG concentrations were significantly higher in sows than in gilts. Colostrum from sows had significantly greater mitogenic activity than colostrum from gilts and this fact can be associated with the potential to accelerate the maturation of a newborn’s gastrointestinal tract. Our findings suggest that parity order may be one among other factors influencing the cell population and, consequently, the immune adaptive response in piglets that induces neutralizing antibodies and cellular immune responses to antigens.

## Introduction

Newborn piglets go from a sterile or extremely low density intrauterine microbiome environment to an antigen-rich external environment, so that they need an adequate immunologic response to survive [1,2]. At birth, piglets have very limited body reserves [1] and, due to the epitheliochorial structure of the placenta, they don’t receive antibodies prenatally [3]. As such, they are born agammaglobulinemic, with limited cell-mediated immunity and no effector and memory T lymphocytes. Therefore, piglets are extremely dependent on the acquisition of maternal immunity via colostrum. During this period, immunity passively transferred through colostrum is crucial in the interval between exposure to pathogenic microorganisms and development of an effective immunologic response.

Immunomodulatory and antimicrobial factors, including antibodies and a variety of cells, are integral parts of colostrum [3–5]. It is thought that immunoglobulin G (IgG) is concentrated from the blood to colostrum in the duct gland via a neonatal Fc receptor (FcRn) dependent mechanism [6]. Also, lymphocytes derived from the common mucosal system migrate to the duct gland and may be found in colostrum [7].

It has been documented that colostrum yield and composition are often influenced by various characteristics of the sow and litter, including dam parity order [8]. In general, IgG concentrations in colostrum at parturition are also altered by parity; IgG concentrations in sows with >3^rd^ parity are greater when compared to first parity sows 24 h postpartum [9,10].

Several authors have investigated the cellular composition of colostrum [11–14] and milk [15]; but, to the best of our knowledge, the evaluation of the major lymphocyte subsets in swine colostrum have not been entirely evaluated. The T cell population in colostrum has been investigated in some studies [4,16–18]. Colostrum is composed of a high number of leukocytes, mainly macrophages and neutrophils. Neutrophils are thought to play a principal role in protecting the sow instead of contributing to the development of the piglets’ immune system. Since the primary report about the presence of both CD3^+^/CD4^+^ (helper/inducer) and CD3^+^/CD8^+^ (cytotoxic/suppressor) T cells in colostrum, T lymphocytes reflect the physiologic and immunologic conditions of the sow and could be involved in the early immunologic response of piglets [5,12–15,19]. However, attention should be paid to the fact that T lymphocytes failed to randomly accumulate in colostrum but rather their presence is the result of a selective homing process. In vitro proliferation assays have shown that T lymphocytes in colostrum respond selectively to enteric microbial antigens [20]. Some research groups indicate that maternal lymphocytes from colostrum are functional and have antigen-specific activity in some organs, because the cells are able to cross the intestinal epithelial barrier of neonatal piglets and migrate via blood to peripheral tissues, such as the spleen, liver, lungs and lymph nodes [16,21]. The relationship between the presence of B cells and their subsets in colostrum and milk is practically unknown. The mammary duct gland contains an extravascular population of B lymphoblasts, precursors of the immunoglobulin plasma cells, which plays a key role in the passive protection of neonates by secreting immunoglobulins into colostrum and milk [22,23].

Cell-mediated immunity is an important contributing factor for disease control of colostrum immune cells that has been overlooked in favor of non-cellular factors such as immunoglobulins [4]. Regarding mammary secretions, the cell types found vary within the species; in swine, colostrum cells are composed mainly of polymorphonuclear cells [24] and to a lesser extent, lymphocytes (B and T cells). Passively transferred lymphocytes from sows to offspring reach the circulatory system and can proliferate and participate in *Mycoplasma hyopneumoniae* specific immunity in piglets [18]. Cell-mediated immunity has been investigated in neonate calves vaccinated against tetanic toxoid and treated with different colostrum sources. The authors observed that colostrum leukocytes are beneficial to neonate T cell responses [25].

Hence, this study aimed to compare immunoglobulin content and immune cell population in the colostrum of gilts and sows right after farrowing and to characterize their subsets of lymphocytes and mitogenic activity.

## Materials and methods

### Animals

This study was approved by the Ethical Committee for Animal Experimentation of Embrapa Suínos e Aves (protocol No. 001/2016). The experiment was conducted on 80 crossbred Landrace 3Large White (LR3LW) dams, being 40 gilts and 40 sows (third and fourth parity). During gestation, sows were housed individually on a slatted floor. Sows were weighed just before being transferred to the farrowing room one week before the expected farrowing date. Sows had free access to water and were fed twice a day on a traditional gestation diet. The gestation diet was provided until the second day of lactation.

### Farrowing management and sample collection

The farrowing process was induced by injection of an analogue of prostaglandin F2a (Alfabédyl®, CEVA Santé Animale, Libourne, France, 1 mL intramuscular) on the 113^th^ day of gestation, except when parturition had already started or was imminent. Oxytocin was not administered during parturition since it interferes with mammary secretion. Colostrum was manually collected into sterile 50-mL conical tubes from all functional teats up to a final volume of 25–30 mL, just after the birth of the first piglet. In order to minimize colostrum contamination, teats were previously scrubbed with iodine alcohol and handling was performed wearing disposable latex gloves.

All sows were evaluated to exclude the possibility of puerperal disorders, mainly mastitis-metritis-agalactia syndrome. Thus, rectal temperature postpartum (> 39.5°C), feed consumption, cell count (> 10^7^/mL), and colostrum pH (> 6.7) were measured [26,27]. All animals were then housed for use in further research.

### Cell viability measurement

Cell suspensions were counted using an automated Coulter counter (Orflo Moxi Z, USA) and analyzed by a single operator using a Neubauer hemocytometer. Each sample was mixed with a 0.4% trypan blue (Sigma Chemical Co., Germany) solution at a ratio of 1:2 (v/v). Cell concentration corresponded to the average of all four sets of squares evaluated considering the volume of the Neubauer chamber and dilution. Trypan blue stained cell counts were used to determine the concentration of non-viable cells. The proportion of non-viable cells was calculated based on the number of trypan blue stained cells (non-viable) compared to total cells.

### Preparation of cells for flow cytometry

Colostrum samples were diluted 1:3 (v/v) in phosphate-buffered saline (PBS; Gibco) containing 5% fetal bovine serum (FBS; Sigma-Aldrich). They were centrifuged for 20 min (1300xg at room temperature) and the upper fat layer was discarded afterwards [12]. Cell densities were calculated and adjusted to 2 x 10^7^ cells/mL, and 50 μL was transferred to wells of a 96-well round-bottom microtiter plate (approximately 1–2 x 10^6^/well).

### Cell staining with antibodies

Antibodies raised against porcine leukocyte antigens were purchased from BioRad Serotec (Oxford, UK), and the stabilizing fixative, FACSLyse and compensation beads were purchased from BD (North Ryde, Australia). The flow cytometry buffer was prepared in PBS supplemented with heat inactivated FBS (2% v/v), bovine serum albumin (2% w/v, Sigma-Aldrich) and sodium azide (0.01% w/v, Sigma-Aldrich). Cells were then treated with 10% (v/v) normal mouse serum to block unoccupied binding sites on secondary antibodies and stained with specific monoclonal antibodies (mAb), which were chosen according to Dawson and Lunney [28]. Despite the high homology for some orthologous proteins, there is still uncertainty in their nomenclature [28]. Therefore, these clusters are named as swine workshop clusters (SWC) and their CD marker orthology is in parentheses.

Colostrum cells (suspended in a flow cytometry buffer at approximately 1 x 10^6^ cell/mL) were incubated for 30 minutes at room temperature with a cocktail of specific mAb. Our ultimate goal was the development of a 4-color flow cytometry panel to assess major lymphocyte, monocyte, granulocyte and NK cell populations. The following fluorochrome-labeled mAbs were used: panel A): 7-AAD (BD Biosciences); panel B): FITC-granulocyte (clone 6D10), RPE-CD79a (clone MB-1), PE-Cy7-CD3 (clone PPT3), APC-macrophages (clone BA4D5); panel C): FITC-CD45RA (clone MIL13), RPE-CD4alpha (clone MIL17), PE-Cy7-CD3 (clone PPT3), APC-SWC2 (or CD27, clone B30C7); panel D): FITC-CD45RA (clone MIL13), RPE-CD8alpha (clone MIL12), PE-Cy7-CD3 (clone PPT3), APC-SWC2 (or CD27, clone B30C7); panel E): FITC-CD4alpha (clone MIL17), RPE-CD8alpha (clone MIL12), PE-Cy7-CD3 (clone PPT3), APC-CD335 (clone VIV-KM1); panel F): FITC-IgM (clone AAI48), RPE-CD45RA (clone MIL13), PE-Cy7-SWC7 (or CD19, clone CC55); panel G): FITC-CD5 (clone 1H6/8), PE-Cy7-SWC7 (or CD19, clone CC55); and panel H): FITC-CD14 (clone MIL2), RPE-CD16 (clone G7), APC-macrophages (clone BA4D5). To evaluate fluorochrome unspecific staining, isotype controls for anti-IgG1, anti-IgG2a and anti-IgG2b were introduced in the preliminary procedure to set up photomultiplier and instrument technical parameters (Table 1). Antibody dilution for the experiment was established through previous titration (Table 1).

**Table 1:** Antibodies used in flow cytometry.

Antibody	Species	Clone	Fluorochrome	Labeling strategy	Isotype	Concentration (mg/mL)	Working dilution
7-AAD	NA	NA	7-AAD	Dye	NA	0.1	1/400
granulocyte	mouse anti-pig	6D10	FITC	Directly conjugated	IgG2a	0.1	Neat
CD45RA	mouse anti-pig	MIL13	FITC	Directly conjugated	IgG1	0.1	Neat
CD4alpha	mouse anti-pig	MIL17	FITC	Directly conjugated	IgG2b	0.1	Neat
IgM	goat anti-pig	AAI48	FITC	Directly conjugated	IgM	1	1/100
CD5	mouse anti-pig	1H6/8	FITC	Directly conjugated	IgG2a	0.1	Neat
CD14	mouse anti-pig	MIL2	FITC	Directly conjugated	IgG2b	0.1	10-Jan
CD79a	mouse anti-human	HM57	RPE	Directly conjugated	IgG1	Not determined	10-Jan
CD4alpha	mouse anti-pig	MIL17	RPE	Directly conjugated	IgG2b	Not determined	1/100
CD8alpha	mouse anti-pig	MIL12	RPE	Directly conjugated	IgG2a	Not determined	1/100
CD45RA	mouse anti-pig	MIL13	RPE	Directly conjugated	IgG1	Not determined	10-Jan
CD16	mouse anti-pig	G7	RPE	Directly conjugated	IgG1	Not determined	10-Jan
CD3	mouse anti-pig	PPT3	RPE-Cy7^a^	Secondary antibody^a^	IgG1	0.1	10-Jan
SWC7 or WC4	mouse anti-bovine	CC55	RPE-Cy7^a^	Secondary antibody^a^	IgG1	0.1	Neat
macrophages	mouse anti-pig	BA4D5	APC^b^	Secondary antibody^b^	IgG2b	0.1	Neat
SWC2 or CD27	mouse anti-pig	B30C7	APC	Directly conjugated	IgG1	Not determined	10-Jan
CD335	mouse anti-pig	VIV-KM1	APC	Directly conjugated	IgG1	Not determined	1/100
IgG1 isotype control	mouse	NA	FITC	Directly conjugated	IgG1	0.1	10-Jan
IgG2a isotype control	mouse	NA	FITC	Directly conjugated	IgG2a	0.1	10-Jan
IgG2b isotype control	mouse	NA	FITC	Directly conjugated	IgG2b	0.1	10-Jan
IgG1 isotype control	mouse	NA	RPE	Directly conjugated	IgG1	Not determined	10-Jan
IgG2a isotype control	mouse	NA	RPE	Directly conjugated	IgG2a	Not determined	10-Jan
IgG2b isotype control	mouse	NA	RPE	Directly conjugated	IgG2b	Not determined	10-Jan
IgG1 isotype control	mouse	NA	APC	Directly conjugated	IgG1	Not determined	10-Jan
IgG1 isotype control	mouse anti-pig	NA	RPE-CY7^a^	Secondary antibody^a^	IgG1	0.1	Neat

*a* MAb dilution following PE-Cy7 labeling (Serotec).

*b* MAb dilution following APC labeling (Serotec).

NA, not applicate.

### Surface and intracellular immunophenotyping

The portions of the sample to be surface-labeled were incubated for 30 minutes at room temperature with conjugated mAb specific for a surface antigen or an irrelevant isotype-matched monoclonal antibody, conjugated to a different fluorochrome. Each case was evaluated by using a panel of monoclonal antibodies. For panels with intracellular staining, cells were resuspended in Cytofix/Cytoperm solution (BD Biosciences) and allowed to sit for another 20 min. Samples were then washed twice with BD Perm/Wash (BD Biosciences) to keep cells permeabilized in order to favor staining of CD79a (the epitope recognized by the mAb is located in the cytoplasmic domain) and CD3 (clone PPT3 recognizes both an extra- and intracellular epitope on CD3) in the subsequent incubation.

After staining, cells were centrifuged (400xg at 10°C for five minutes) and therefore the pellet was washed once with 1 mL of flow cytometry buffer followed by centrifugation (400xg at 10°C for five minutes). The cells were resuspended in 300 μL of stabilizing fixative and transferred to a plate. The samples were analyzed by flow cytometry, which was performed within 2 hours.

### Flow cytometry

**Fig 1:**
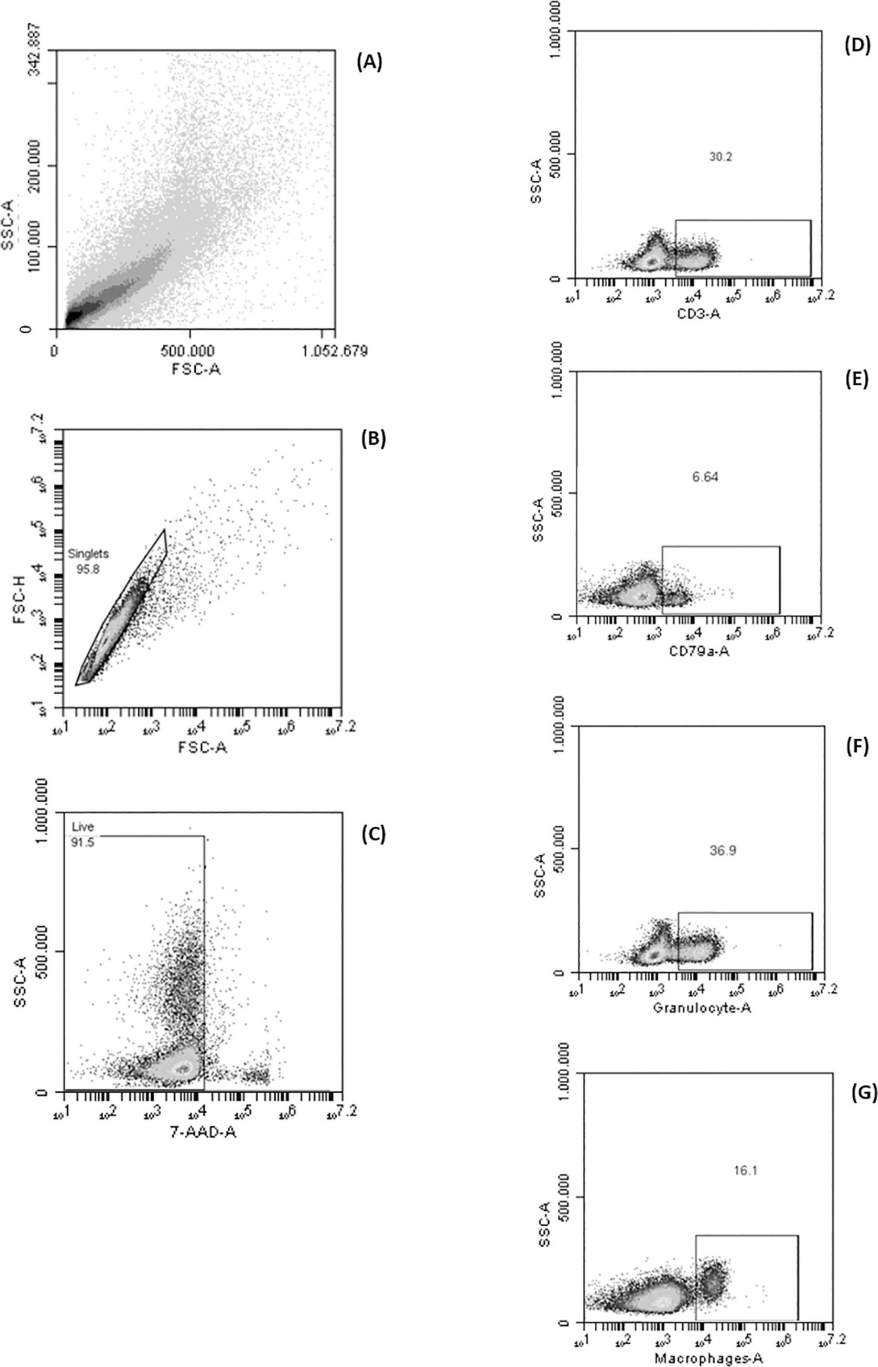
Flow cytometry gating strategy. Cells were first gated based on size (FSC-A) and granularity (SSC-A) to exclude sub-cellular debris (A), then sequentially using FSC-H vs. FSC-A to eliminate doublets (B). The 7-AAD stain was used to exclude cells with damaged membranes (C). Subsequent analysis involved gating according to the desired analysis, for instance, on CD3^+^(D), CD79a^+^(E), granulocytes^+^(F) and macrophages^+^(G).

Flow cytometry was performed on Accuri C6 cytometer (BD Biosciences). Fifty thousand events were analyzed (based on FSC and SSC) using Accuri C6 plus software (Becton Dickinson). Before sample analysis, the flow cytometer settings were checked using Cytometer Setup and Tracking beads (CS&T beads, BD) as described at the manufacturer’s instructions. Compensation beads were used with single stains of every antibody to establish the compensation settings. The compensation matrix was identically applied to all samples. The side scatter (SSC) threshold level was set at 8,000 units to eliminate debris. Gates considered to indicate positive and negative staining cells were set based on fluorescence minus one (FMO) tests of colostrum samples and these gates were carried out systematically on each sample, allowing minor adjustments for SSC variability. Subsequent orientation and specific gates for other populations (such as macrophages, lymphocytes and neutrophils) were identified in colostrum using this gating strategy (Fig 1).

### IgG and IgA quantitation

ELISAs were performed to quantitate IgG and IgA immunoglobulin concentrations in colostrum as previously mentioned [29]. Prior to the analysis, colostrum samples were centrifuged (1,300xg at 4°C for 20 min) to remove fat. All colostrum samples were diluted 1:500,000 (v/v) with proper diluent (50 mM Tris buffer, 0.14 M NaCl, 1% BSA, and 0.05% Tween 20). ELISA reagents were obtained from Bethyl Laboratories (Montgomery, TX, USA). Briefly, 100 μL of colostrum sample or standard solution was added to each well and incubated at room temperature for 1 hour, then washed four times with the washing buffer. The concentrations of IgG and IgA in the standard solutions were 333.3, 111.1, 37, 12.3, 4.1, 1.37, and 0 ng/mL. All samples were analyzed in duplicate.

Afterwards, 100 μL of either anti-IgG or IgA was added and incubated at room temperature for one hour and washed four times with the washing buffer. Then 100 μL of horseradish peroxidase was added. The plates were incubated for 30 min at room temperature and were washed four times with the washing buffer. The TMB (3,3′,5,5′-tetramethylbenzidine) substrate solution was added to the plates and incubated for 30 min in the dark. The reaction was terminated by adding 100 μL of stop solution. The plates were read on a microplate reader (Thermolab System, MRX Revelation, Chantilly, VA) at 540 nm. The results were obtained in ng/mL but expressed in mg/mL after appropriate dilution factor correction.

### Cell culture

The rat intestinal epithelial crypt cell line (IEC-6, BCRJ Cat#0117, RRID:CVCL_ 0343) was obtained from Banco de Células do Rio de Janeiro (BCRJ, Rio de Janeiro, Brazil). Cells (passage 12 and mycoplasma negative) were cultured at 37°C in a humidified atmosphere of 5% CO_2_ in Dulbecco’s Modified Eagle Medium (DMEM) (Gibco-BRL, Life Technologies) supplemented with 4 mM L-glutamine adjusted to contain 1.5 g/L sodium bicarbonate and 4.5 g/L glucose and supplemented with 0.1 Unit/mL bovine insulin (Sigma-Aldrich), and 10% FBS (Gibco-BRL, Life Technologies).

### Mitogenic assay

The use of carboxyfluorescein diacetate succinimidyl ester (CFSE) in combination with 7-AAD enabled the concomitant determination of cell proliferation. IEC-6 cells were suspended (1×10^6^ cells/mL) in DPBS and labeled with CFSE (2.5 μM; Molecular Probes, USA) for ten minutes at 37°C. The labeling process was stopped by the addition of five volumes of ice-cold RPMI 1640 containing 10% FCS (RPMI-FCS) followed by incubation for five minutes in ice, protected from light. The cells were washed two times with 20 mL of DMEM-FBS and further suspended in the same medium. The cells were plated in 24-well plates (1×10^4^ cells/well), allowed to adhere for 18 h and were then washed twice in Hank’s Balanced Salt Solution (HBSS) (Sigma-Aldrich) [30]. The media was then changed to 1 mL of DMEM without serum and cultured with colostrum (100 μL). As positive control, two wells received cell culture with FBS (100 μL), and cell culture without stimulants represented the negative control (untreated). The cells were cultured for 48 h at 37°C under 5% CO_2_. The cells (1×10^5^) were transferred to flow cytometry tubes and labeled with 7-AAD. A total of 50,000 events per tube was acquired on the flow cytometer (Accuri C6plus and FACSCanto, Becton-Dickinson, USA) and analyzed. Cells were recovered after cultivation for 48 hours and evaluated for CFSE staining intensity.

The percentage of proliferated IEC-6 cells was determined by CFSE dilution and the geometric mean values of the colostrum-stimulated triplicates were calculated and divided with the geometric mean values of the medium control triplicates in order to obtain the stimulation index (SI).

### Statistical analysis

The statistical methodology applied was Analysis of Variance, using the MIXED procedure of SAS [31], testing the treatment effect. The likelihood ratio test indicated significant heterogeneous error variances between treatments, modeling them using the GROUP option of the REPEATED statement. Data are expressed as the mean percentage of positive cells ± standard deviation (SD). Significance was declared at p ≤ 0.05. Colostrum cell concentrations from the same sow were examined using the Friedman test and these categorical data were summarized using frequency distributions.

## Results

### Analysis of the immune components in porcine mammary secretions by flow cytometry

**Fig 2:**
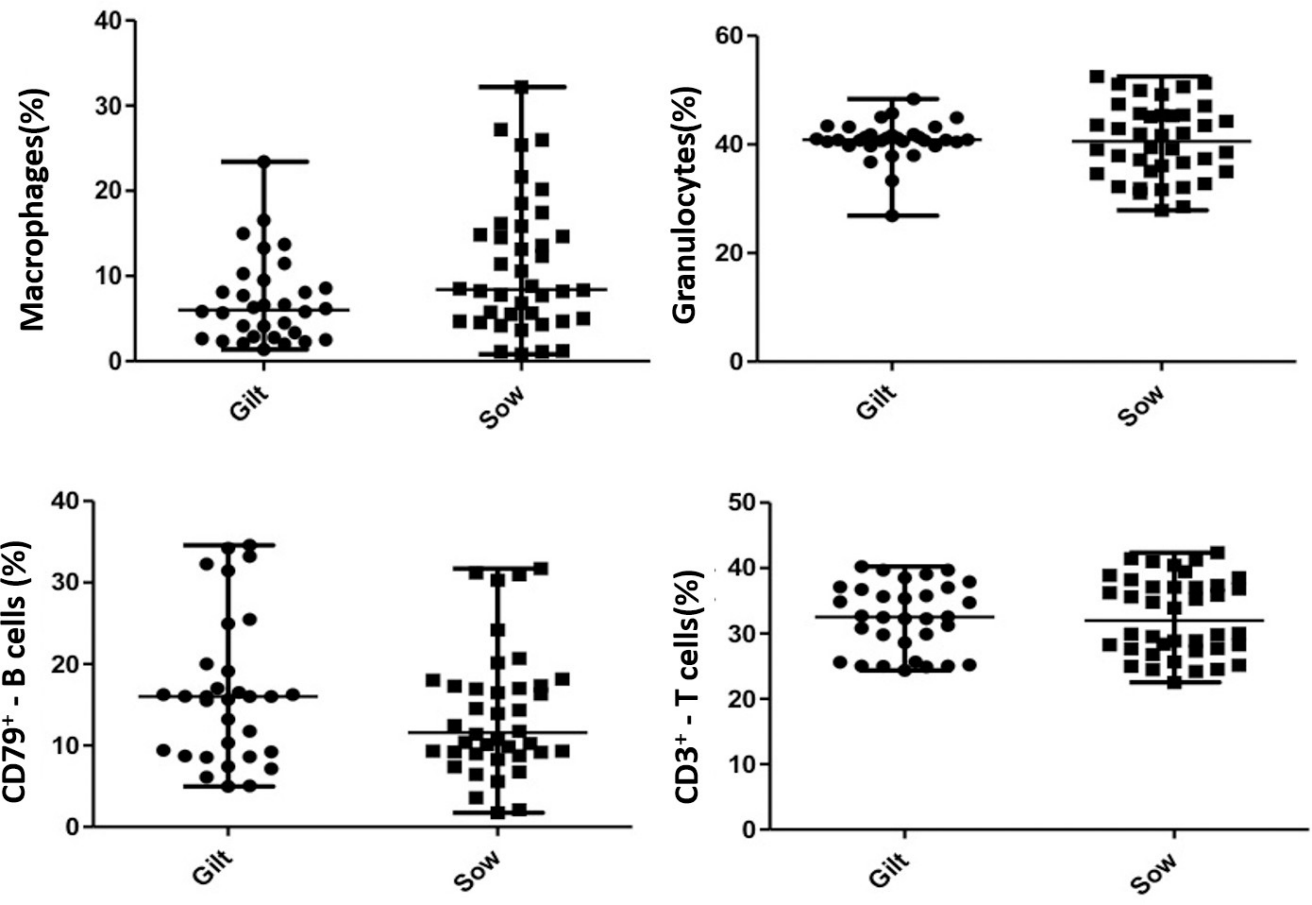
Immune cells present in colostrum from gilts and sows. The line shows the median value for each colostrum sampling point. Data shows concentrations of total Macrophages^+^, Granulocytes^+^, B cells (CD79a^+^) and T cells (CD3^+^) in colostrum; no difference found between groups.

Trypan blue exclusion staining and microscopic evaluation of viability were performed right after colostrum collection and  at  subsequent moments, according to the flow cytometric analysis. The evaluated colostrum were rich in cells (5x10^6^ to 8x10^6^ cells/mL for gilts and sows, respectively), and their cell viability was greater than 95% [5,15]. A summary of the cellular components in the colostrum of gilts and sows is shown in Fig 2. In both categories of dams, the foremost predominant immune cell types were granulocytes (neutrophils, 40%) followed by T lymphocytes (CD3^+^, 30%), B lymphocytes (CD79a^+^, 13–16%) and macrophages (7–11%). The phagocytic cell population of mammary secretions consisted of neutrophils and macrophages.

The gate to identify T lymphocyte and NK cell populations was not based on forward scatter (FSC) and side scatter (SSC) properties, but rather they were used for estimating lymphocyte population and exclusion of debris [17]. Doublet exclusion was performed by plotting the forward scatter height (FSC-H) against the forward scatter area (FSC-A). We also excluded dead cells using 7-AAD staining, a single parameter histogram to identify dead cells which were positive for the dye. Then, CD3^+^ cells were considered to represent 100% of T lymphocytes. Among T lymphocytes, CD3^+^CD8^+^ cells (47–49%) were more numerous than CD3^+^CD4^+^ cells (7–14%) (Table 2). Interestingly, CD3^+^CD4^+^CD8^+^ double-positive cells (*p* = 0.0243), and CD4^+^ cells were significantly higher in sows in relation to gilts. The phenotypic classification of CD4^+^ T cell subsets that rose in sows were naive CD4^+^ T cells (CD4^+^CD27^+^CD45RA^+^) (*p* = 0.0408), central memory CD4^+^T cells (CD4^+^CD27^+^CD45RA^-^) (*p* = 0.0089), and effector memory CD4^+^T cells (CD4^+^CD27^-^CD45RA^-^) (*p* = 0.089). Overall, sows tended to show an increased percentage of CD4^+^ cells when compared to gilts. In relation to CD8^+^ cells, sows presented higher content of central memory CD8^+^ (CD8^+^CD27^+^CD45RA^-^) (*p* = 0.0156) in colostrum in comparison to gilts. Memory T cell (CD4^+^CD27^+^CD45RA^-^, CD4^+^CD27^-^CD45RA^-^ and CD8^+^CD27^+^CD45RA^+^) phenotypes predominated among this subset, as indicated by the higher proportion of T cells in colostrum.

**Table 2:** Percentages of cells (T lymphocyte, B lymphocyte, and monocyte subsets) in colostrum from gilts and sows.

Immune Cells (%)	Dam Category		
	Gilt	Sow	*P*—value
***B cells (CD79a***^***+***^***)***			
SWC7^+^IGM^+^	18.93±1.085	25.63±4.022	0.1274
SWC7^+^CD45RA^+^	1.856±0.547	2.846±0.470	0.1866
SWC7^+^CD5^+^	5.219±1.251^a^	10.72±1.535^b^	0.0209
SWC7^+^CD5^-^	23.18±1.103	26.33±0.963	0.0581
***T cells***			
**CD3**^**+**^	32.36±1.820	32.53±1.884	0.9500
**CD4**^**+**^	9.354±2.277	11.81±2.117	0.1866
CD27^+^CD45RA^-^	8.380±1.215^a^	18.31±3.110^b^	0.0089
CD27^+^CD4RA^+^	2.95±0.761^a^	6.25±1.274^b^	0.0408
CD27^-^CD45RA^-^	21.53±2.360^a^	32.16±3.331^b^	0.0089
**CD8**^**+**^	47.205±1.536	47.880±1.266	0.7366
CD27^+^CD45RA^-^	10.09±1.706^a^	18.33±2.348^b^	0.0156
CD27^+^CD45RA^+^	3.84±1.077	7.42±2.050	0.1418
**CD3**^**+**^**CD4**^**+**^**CD8**^**+**^	14.96±1.591^a^	22.10±1.97^b^	0.0243
***Myeloid cells***			
**Macrophages**^**+**^	7.062±1.589	11.06±2.408	0.2092
Macrophages^+^CD16^+^	27.60±1.286	25.90±1.945	0.4996
Macrophages^+^CD14^+^CD16^+^	10.88±1.695^a^	16.76±1.888^b^	0.0380
***NK cells***			
**CD3**^**-**^**CD8**^**low**^**CD335**^**+**^	13.20±1.098	16.31±1.735	0.1732

The analysis was performed with the Kruskal-Wallis tests with Dunn’s multiple comparison post-test.

^a, b^ Different superscript letters indicate significant statistical difference between groups (p ≤ 0.05).

Cell populations in colostrum: conventional B cells: SWC7^+^CD5^–^ and SWC7^+^CD5^+^; naive B cells: SWC7^+^IgM^+^; mature B cells: SWC7^+^CD45RA^+^; naive CD4^+^ T cells: CD4^+^CD27^+^CD45RA^+^; central memory CD4^+^T cells: CD4^+^CD27^+^CD45RA^-^; effector memory CD4^+^T cells: CD4^+^CD27^-^CD45RA^-^; naive CD8^+^ T cells: CD8^+^CD27^+^CD45RA^+^; central memory CD8^+^T cells: CD8^+^CD27^+^CD45RA^-^; NK cells: CD3^-^CD8^low^CD335^+^.

B lymphocyte subset distribution is indicated in Table 2. The gate is similar to T lymphocyte, although B lymphocyte was defined as the sum of CD79a^+^. Singlet cells were gated according to FSC-A × FSC-H dispersion, and then mononuclear cells were gated according to FSC-A × SSC-A dispersion (Fig 1). Live cells were gated as those negative for the 7-AAD cell viability marker. The gate for positive cells to CD79a^+^ was determined as the cells located on the right side of the unstained cells. The proportion of naive B cells (SWC7^+^IgM^+^) and mature B cells (SWC7^+^CD45RA^+^) was similar between the gilt and sow groups, while, on the contrary, SWC7^+^CD5^+^ cells were higher in sows than in gilts (*p* < 0.05, Table 2).

Monocytes/macrophages (macrophages^+^CD14^+^CD16^+^) were also significantly higher in sows (16.76% *vs* 10.88% in gilts) (Table 2). This population was analyzed by first gating using FSC and SSC. Other numerical differences (not statistically significant) were found in NK cells (CD3^-^CD8^low^CD335^+^) in sows (16%) when compared to gilts (13%).

### Humoral analysis

**Fig 3:**
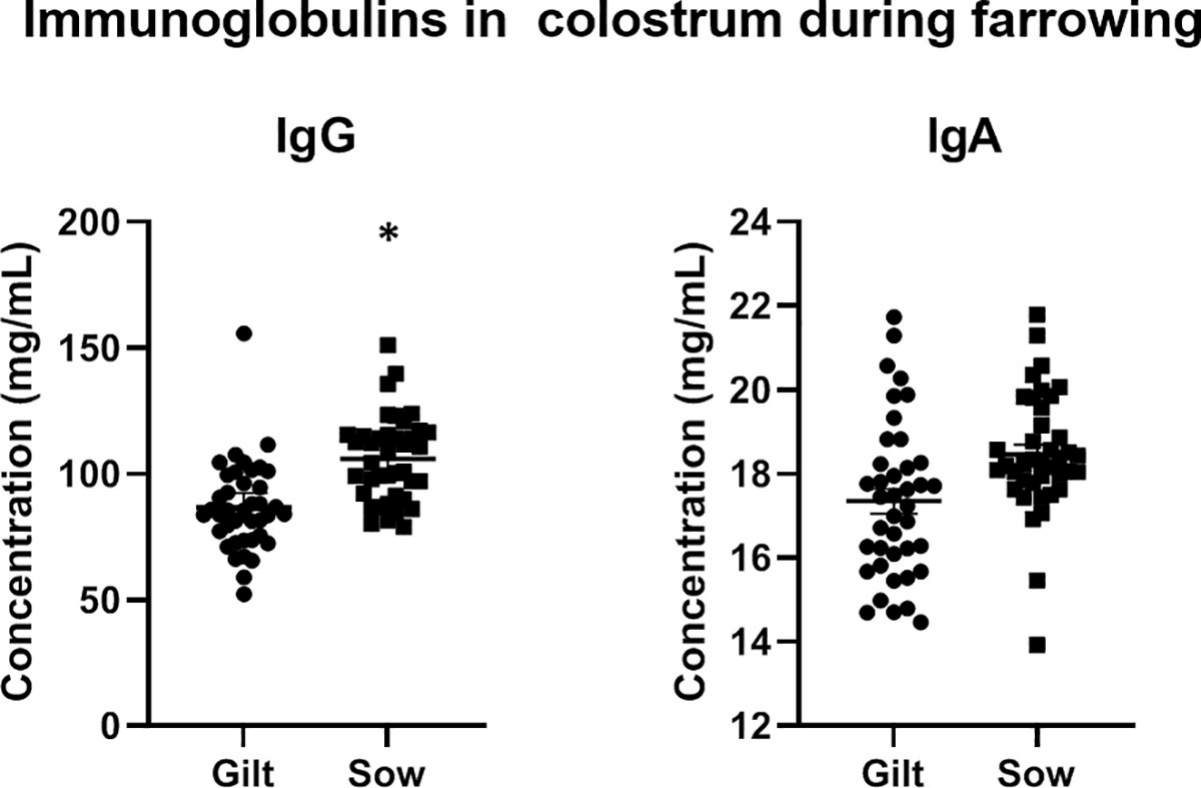
Comparison of total IgG and IgA concentration (mg/mL) in colostrum from gilts and sows. Error bars represent the standard error of the mean (± SEM). Gilt IgA (17.5± 1.8) vs. Sow IgA (18.3± 1.4), *p* < 0.1650; *Gilt IgG (90.0± 21.3) vs. Sow IgG (102.9± 21.6), *p* < 0.0112.

IgA concentrations did not differ between gilts and sows (Fig 3). As expected, IgG concentrations were significantly higher in sows than in gilts (*p* = 0.011).

### Cell proliferation analysis

**Fig 4:**
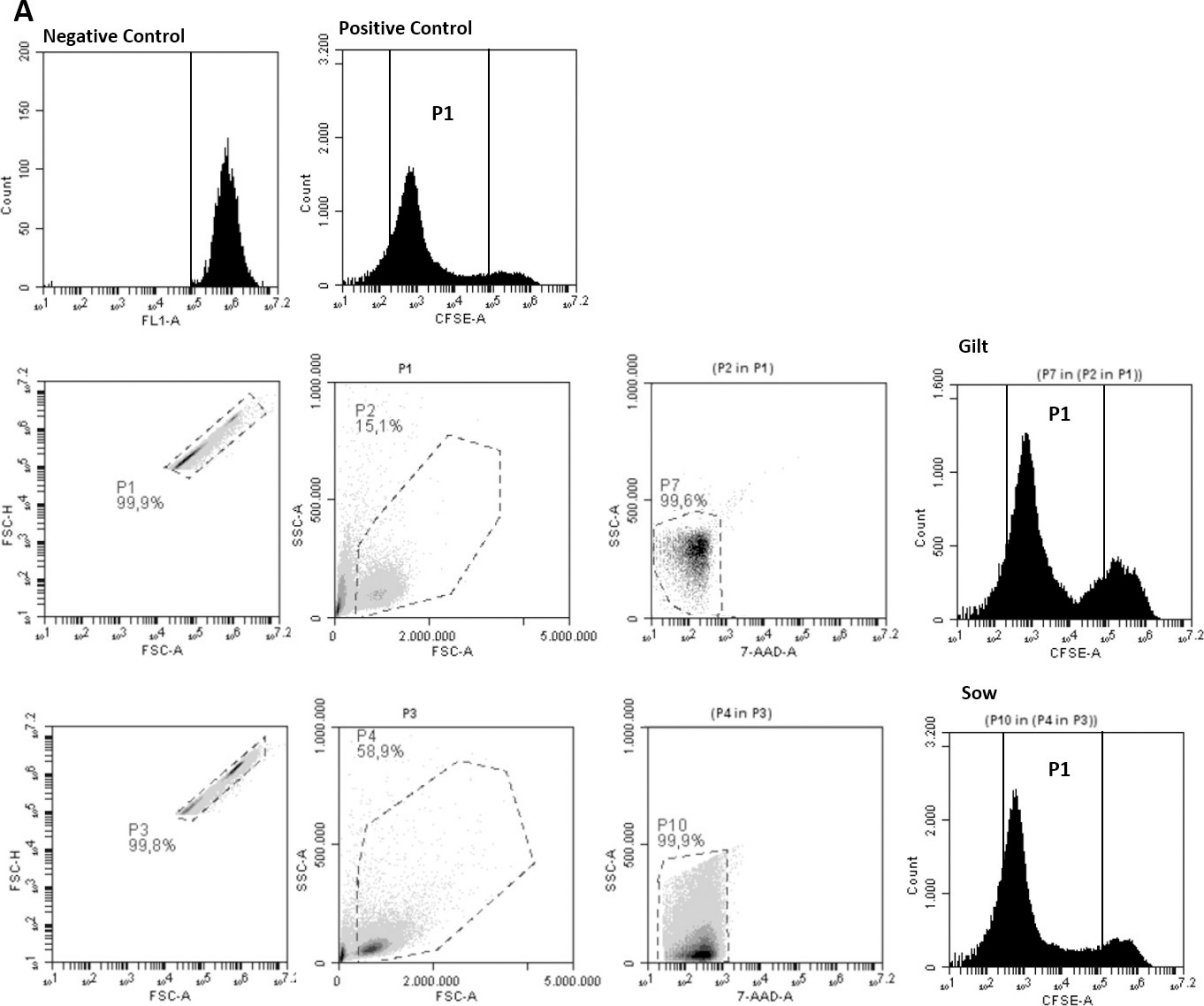
Dot plots and histograms representative of an IEC-6 proliferation assay using CFSE. IEC-6 cells (1x10^6^ cells/mL) were labeled with CFSE (2.5 μM) and cultured in 24-well plates with colostrum (100 μL) from sows and gilts, positive control with added FBS, and negative control without FBS. Cells were recovered after cultivation for 48 hours and evaluated for CFSE staining intensity. Live, singlet cells were gated using forward scatter properties (FSC-H and FSC-A). The P1 regions represent the IEC-6 cell division profile compared with negative control.

**Fig 5:**
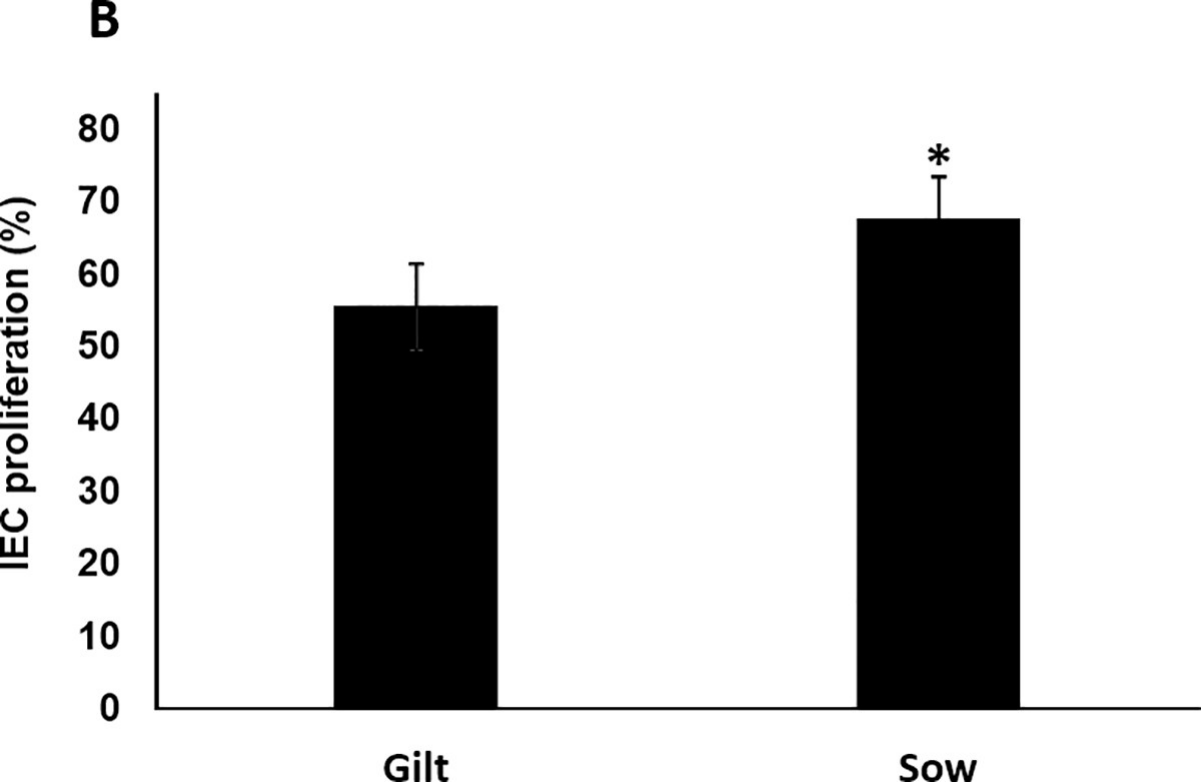
Mitogenic activity of colostrum from gilt and sow on intestinal epithelial cells (IEC-6). Data are expressed as the mean (± SEM) percentage of cell viability calculated in relation to untreated cells (negative control). Gilt (55.45± 9.74) vs. Sow (67.47± 6.76), *p < 0.001.

The cell proliferation showed a higher rate in IEC-6 cells stimulated with colostrum from sows than from gilts at 48 h. Although all colostrum samples stimulated proliferation of IEC-6 compared to the control, colostrum from sows had significantly greater (*p* < 0.001) mitogenic activity than colostrum from gilts (Figs 4 and 5).

## Discussion

Although differences in porcine colostrum are reported for immunoglobulins, the understanding and the effects of immune cell subsets are still limited. The proportions of immune cells, which are present in porcine colostrum are also influenced by several factors, such as season, parity, genotype and vaccination. These factors may explain the differences in cells and immunoglobulins [14,24,32]. Based on that, we analyzed the relationship between sow parity stage and quantity of each type of immune cell observed in colostrum. The effects of parity order or age in colostrum cell characteristics have been documented in other species. In bovines, heifers had significantly higher mitogen- and antigen-induced proliferation of their colostral leukocytes than third parity or older cows [33]. In another study, percentages of CD4^+^ and CD4^+^CD26^+^ lymphocytes and the CD4^+^/CD8^+^ ratio were significantly lower in heifers than in >3^rd^ parity cows, suggesting that mammary gland immune cells are more active in cows with higher parity compared to heifers [34]. In an investigation conducted in humans, parity was positively correlated to the concentration of neutrophil-macrophage cells (t = -2.07, r = 0.205) and negatively correlated to lymphocyte-plasma cells (t = 2.073, r = -0.101) [35].

Our data indicates that about 32% of lymphocytes were T lymphocytes and about 15% B lymphocytes. This finding shows that the major lymphocyte subpopulation in colostrum were CD3^+^ cells, which is in line with results obtained by Le Jan [12] and Pomorska-Mol, Markowska-Daniel [13] in swine species, as well as in bovines by Park, Fox [36] and Yang, Ayoub [37]. Le Jan [12] showed that the percentages of most leukocyte subpopulations were higher in sow colostrum. Although some investigators claimed that parity plays a key role during this process, it is interesting to note that our outputs for macrophages, lymphocytes and granulocytes suggest no parity effects.

Colostrum T lymphocytes expressing activation markers are a definite cell population that shows phenotypical differences from those found in peripheral blood [12,13]. They express in vitro functional capabilities, e.g. response to bacterial and viral antigen stimulation. Our results show that T lymphocyte subsets are composed of helper and cytotoxic subsets, mainly CD3^+^CD8^+^, which are documented to be cytokine-producing cells, mainly providing proinflammatory mediators [12,13,17]. It must be observed, however, that our findings are consistent and corroborate with previous studies that studied the content of T lymphocyte subsets, characterized by the expression of activation markers, in the colostrum of pigs and their wide variation between gilts and sows [13,15,17]. The major T cell subset in porcine blood and various other lymphatic and non-lymphatic organs are lymphocytes CD3^+^CD4^+^CD8a^+^ or double-positive cells. The latter corresponds to the T cell developmental stage within the thymus. We showed that this population is higher in sow colostrum. In fact, double-positive lymphocyte cells exhibit properties of mature antigen-experienced cells, and porcine CD4 T cells up-regulate CD8alpha after antigen encounter. In particular, a huge proportion of CD4 T cells in blood expresses CD8alpha in older pigs, suggesting that these cells could include memory/effector T cells [17,38–42] and that porcine CD4^+^T cells can express CD8alpha and the frequency of CD4^+^CD8alpha^+^T cells increases with the progression of age [41,43,44].

T cells adhere and cross the gastrointestinal mucosa, reaching the blood circulation, where they continue to be immunologically active. Additionally, in humans as an example, immune memory cells (CD45RO^+^) have been isolated in feces from infants fed with human milk [45]. The porcine CD45RA/RC expression probably represents a marker of naive and terminally differentiated T cells [46]. Besides, our findings showed that colostrum contains memory T lymphocytes (central memory CD4^+^/CD8^+^ T cells and effector memory CD4^+^T cell) to safeguard the piglets via the sow’s previous immune experience and to contain high levels of T lymphocytes. Some studies suggested that these T lymphocytes that produce cytokines actively defend the offspring [47,48]. This fact highlights that sows provide higher protection than gilts. Moreover, colostrum can help the postnatal development of both T cell responses. Helper lymphocytes are functionally subdivided according to the pattern of cytokines they produce. During the stimulus provided by an antigen-presenting cell (APC), a precursor Th0 lymphocyte can become a Th1, Th2 or Th17 lymphocyte, depending on the cytokine environment. Although morphologically indistinguishable, these cells show different patterns of secreted cytokines and consequently different effector responses [47]. Thus, the influence of cytokines present in colostrum should also be taken into consideration, because cytokines have effects on T cells and are produced by different T helpers (Th1/Th2/Th3/Tr1). Nevertheless, these cytokines have not been clearly defined [24,47]. Thus, the transfer of various cytokines in the colostrum of gilts and sows is a promising area for further investigation to better comprehend the mechanisms involved and its benefits.

In our study, B cell proportions were similar between groups, however SWC7^+^CD5^+^ cells were higher in sows. In humans, these cells are called B1 cells, but this cell population has not been characterized in pigs yet [49]. Further evaluations, however, are needed to support this finding of the B1 cell phenotype in swine. Conversely, the odds of conventional B cells (SWC7^+^CD5^-^), the primary (naive) subpopulation of B cells (SWC7^+^IgM^+^), and mature B cells (SWC7^+^CD45RA^+^) did not differ between groups. Our findings showed a presence of effector T and B cells (SWC7^+^CD45RA^+^) [46]. B cells could provide identical protection against enteric pathogens within the digestive tract of a newborn by colostrum intake [49]. Hence, B cells content in colostrum may serve as a selective strategy to guarantee immune defense to the neonate [50].

In agreement with several recent studies, conducted by flow cytometry analysis, differences in expression were revealed between some cell surface markers (CCR7 and CD25) in colostrum and blood natural-killer cells and T cells [17,46,51]. Moreover, the sows’ colostrum presented significantly higher effector memory CD3^+^CD4^+^CD8^+^ double-positive and CD4^+^T lymphocyte (CD4^+^CD27^-^CD45RA^-^) than that of gilts. Ogawa et al. [39] suggested that all or most T cells in colostrum have an effector-like phenotype and thus are more activated than T cells in blood, in addition to the fact that their gene expression profile would enable migration of these T cells to mammary glands, be secreted in colostrum, and likely contribute to passive immunity provided by sows to newborn pigs. Effector T and B lymphocytes can also compensate for the immature function of neonatal T cells and promote their maturation [20].

It has been assumed that gilts may produce colostrum and milk with lower levels of immune factors than sows, due to having less exposure time to pathogens over their lifetime and a naive immune system compared to older sows. Although total immune cells in the current study presented no difference between gilts and sows, IgG content was greater in sows, which is consistent with previous results [9,10,52]. However, our findings about IgA are different from those observed by these studies, but similar to Craig, Dunshea [11]. It has been assumed that multiparous sows may produce colostrum and milk with higher levels of immune factors than gilts due to having had time for exposure to pathogens over their lifetime and a relatively mature immune system compared to gilts [8–10]. However, this was not reflected in total colostrum IgA concentrations in the current study, with no difference between primiparous and multiparous sows, in contrast to several recent studies [9,52]. These differences may be due to responses to nutritional changes [53], as well as genetic advances, and the timing of collection of colostrum samples [54,55].

Immunoglobulins constitute a vital component of the immunological activity found in milk and colostrum. They are crucial to the immunological link that happens when the dam transfers acquired immunity to the offspring. The employment of colostrum or milk as a source of immunoglobulins, whether intended for the neonate of the species or for a specific species, must be considered regarding types of immunoglobulins present in the secretion, the mechanisms by which the immunoglobulins are secreted, and the mechanisms by which the piglets consuming the milk gain immunological benefit [56]. The steadiness of immunoglobulins, since they undergo digestion within the intestine, is a further issue when evaluating the biological value and impact of colostrum immunoglobulins.

It is, therefore, more likely that reduced growth and higher mortality be observed in gilt litters, which could be a consequence of immunologically poor colostrum and milk in gilts compared to sows. In this sense, Carney-Hinklen, Tao [57] showed that serum IgG concentrations were greater in fourth-parity order compared to first parity.

In fact, the composition of immunoglobulins in colostrum, such as IgG and IgA that neutralize pathogens, is more important than the amount of these proteins. Regarding the probability that sows are more hyperimmunized against one or more antigens than gilts, the former consequently show increased immunoglobulin and virus-neutralizing titers in colostrum compared to the latter. As the mechanisms by which immunoglobulins are transferred from dam to piglet become well documented, additional research is required to clarify the mechanisms of action of immunoglobulin and colostrum immune cells. Moreover, further studies should regard the inhibition of complement activation, toxin-neutralizing antibodies, anti-idiotypic neutralization, Fc interaction, and modulation of cytokine responses. In addition, a promising approach could be the establishment of a swine milk bank to supply colostrum for feeding piglets that are developmentally more immature to reinforce their development.

The intestinal epithelium is a tissue with rapid cellular turnover. Any increase of the cell proliferation rate in the crypts will lead to an overall increase in the epithelial cell population that will result in increased villus height [58]. Colostrum produced by sows has significantly higher growth-promoting activity compared to colostrum from gilts. Thus, sow colostrum showed a higher ability to induce intestinal cell proliferation, which can be associated with the potential to accelerate the maturation of the newborns’ gastrointestinal tract. Porcine colostrum contains critical biological components and nutritional factors such as epidermal growth factor (EGF), insulin-like growth factor I and II (IGF-I, IGF-II), and insulin [59–61], exosomes [62], oligosaccharides [63,64], metabolites [65] and bacteria [66]. Some of these components play a possible vital role in stimulating the development of the neonatal immune system, gut closure and establishing intestinal bacterial communities. In fact, the maintenance of functional integrity of the intestinal epithelial barrier requires coordination of mitogenic intestinal processes. Antimicrobial and immunomodulatory factors of colostrum could be able to compensate or change the neonatal innate and adaptive immune system for immature piglets and mitigate environmental infectious pathogens. Consequently, understanding the main components of porcine colostrum is critical for efficient pig production. Surely, the ideal experiment would be performed on the primary culture of piglet duodenal cells, and that is the intention for future studies. In any case, these results are interesting and not previously described.

However, the information about these biological components for swine is still limited.

Overall, colostrum should be encouraged for nutritional reasons, and mainly for its immunological properties. In addition, supporting our results, other recent studies showed that the survival and growth of piglets does not seem to be impacted by the parity order of the biological dam, since piglets that suckled on sows have better development than those that suckled on gilts [67]. Finally, our results suggest that the cell content presented in colostrum can also contribute to piglet immunity in the first period of life and that piglets that suckled on gilts might have increased vulnerability to diseases during this period.

## Conclusions

According to the present study, colostrum contains a special distribution of lymphocyte subsets, suggesting that these cells probably migrate selectively to the milk. B and T lymphocytes in colostrum seem to be enriched with subsets that have effector functions, which combined with innate immunity can be a prompt line of defense to the litter. Therefore, these findings contribute to grasp the link between the immune profile of the colostrum from gilts and sows, opening new insights for further studies on piglet-sow immunity interactions. This study, while confirming the role of effector immunity in colostrum, may provide a further and deeper contribution for the comprehension of neonatal immunity and of piglet-gilt/sow immune relationships through lactation.

## References

[1] Le Dividich J, Rooke JA, Herpin P. Nutritional and immunological importance of colostrum for the new-born pig. J Agr Sci-Cambridge. 2005;143:469–85.

[2] Stinson LF, Boyce MC, Payne MS, Keelan JA. The Not-so-Sterile Womb: Evidence That the Human Fetus Is Exposed to Bacteria Prior to Birth. Front Microbiol. 2019;10. 10.3389/fmicb.2019.00010 31231319 PMC6558212

[3] Salmon H, Berri M, Gerdts V, Meurens F. Humoral and cellular factors of maternal immunity in swine. Dev Comp Immunol. 2009;33(3):384–93. 10.1016/j.dci.2008.07.007 18761034

[4] Bandrick M, Ariza-Nieto C, Baidoo SK, Molitor TW. Colostral antibody-mediated and cell-mediated immunity contributes to innate and antigen-specific immunity in piglets. Dev Comp Immunol. 2014;43(1):114–20. 10.1016/j.dci.2013.11.005 24252519 PMC3902642

[5] Zhang S, Chen F, Zhang Y, Lv Y, Heng J, Min T, et al. Recent progress of porcine milk components and mammary gland function. Journal of animal science and biotechnology. 2018;9:77. 10.1186/s40104-018-0291-8 30377527 PMC6196465

[6] Schnulle PM, Hurley WL. Sequence and expression of the FcRn in the porcine mammary gland. Vet Immunol Immunopathol. 2003;91(3–4):227–31. 10.1016/s0165-2427(02)00294-5 12586485

[7] Harp JA, Moon HW. Lymphocyte Localization in Lymph-Nodes of Swine—Changes Induced by Lactation. Vet Immunol Immunop. 1988;18(3):219–27. 10.1016/0165-2427(88)90066-9 3394255

[8] Farmer C, Quesnel H. Nutritional, hormonal, and environmental effects on colostrum in sows. J Anim Sci. 2009;87(13 Suppl):56–64. 10.2527/jas.2008-1203 18791139

[9] Klobasa F, Butler JE, Werhahn E, Habe F. Maternal-neonatal immunoregulation in swine. II. Influence of multiparity on de novo immunoglobulin synthesis by piglets. Vet Immunol Immunopathol. 1986;11(2):149–59. 10.1016/0165-2427(86)90094-2 3485853

[10] Quesnel H. Colostrum production by sows: variability of colostrum yield and immunoglobulin G concentrations. Animal. 2011;5(10):1546–53. 10.1017/S175173111100070X 22440345

[11] Craig JR, Dunshea FR, Cottrell JJ, Wijesiriwardana UA, Pluske JR. Primiparous and Multiparous Sows Have Largely Similar Colostrum and Milk Composition Profiles Throughout Lactation. Animals: an open access journal from MDPI. 2019;9(2). 10.3390/ani9020035 30691116 PMC6407016

[12] Le Jan C. A study by flow cytometry of lymphocytes in sow colostrum. Res Vet Sci. 1994;57(3):300–4. 10.1016/0034-5288(94)90121-x 7871248

[13] Pomorska-Mol M, Markowska-Daniel I, Bednarek D. Flow Cytometric Analysis of Leukocytes in Porcine Mammary Secretions. B Vet I Pulawy. 2010;54(2):188–92.

[14] Schollenberger A, Degorski A, Frymus T, Schollenberger A. Cells of Sow Mammary Secretions.1. Morphology and Differential Counts during Lactation. J Vet Med A. 1986;33(1):31–8. 3085387

[15] Evans PA, Newby TJ, Stokes CR, Bourne FJ. A Study of Cells in the Mammary Secretions of Sows. Vet Immunol Immunop. 1982;3(5):515–27. 10.1016/0165-2427(82)90017-4 7147696

[16] Nechvatalova K, Kudlackova H, Leva L, Babickova K, Faldyna M. Transfer of humoral and cell-mediated immunity via colostrum in pigs. Vet Immunol Immunop. 2011;142(1–2):95–100. 10.1016/j.vetimm.2011.03.022 21524802

[17] Hlavova K, Stepanova H, Faldyna M. The phenotype and activation status of T and NK cells in porcine colostrum suggest these are central/effector memory cells. Vet J. 2014;202(3):477–82. 10.1016/j.tvjl.2014.09.008 25438731

[18] Bandrick M, Pieters M, Pijoan C, Molitor TW. Passive transfer of maternal Mycoplasma hyopneumoniae-specific cellular immunity to piglets. Clinical and vaccine immunology: CVI. 2008;15(3):540–3. 10.1128/CVI.00466-07 18184823 PMC2268269

[19] Magnusson U, Rodriguezmartinez H, Einarsson S. A Simple, Rapid Method for Differential Cell Counts in Porcine Mammary Secretions. Vet Rec. 1991;129(22):485–90. 10.1136/vr.129.22.485 1781144

[20] Field CJ. The immunological components of human milk and their effect on immune development in infants. The Journal of nutrition. 2005;135(1):1–4. 10.1093/jn/135.1.1 15623823

[21] Williams PP. Immunomodulating Effects of Intestinal Absorbed Maternal Colostral Leukocytes by Neonatal Pigs. Can J Vet Res. 1993;57(1):1–8. 8431798 PMC1263580

[22] de Jesus Rodriguez B, Chevaleyre C, Henry G, Molle D, Virlogeux-Payant I, Berri M, et al. Identification in milk of a serum amyloid A peptide chemoattractant for B lymphoblasts. BMC immunology. 2009;10:4. 10.1186/1471-2172-10-4 19166592 PMC2637234

[23] Peroni DG, Chirumbolo S, Veneri D, Piacentini GL, Tenero L, Vella A, et al. Colostrum-derived B and T cells as an extra-lymphoid compartment of effector cell populations in humans. The journal of maternal-fetal & neonatal medicine: the official journal of the European Association of Perinatal Medicine, the Federation of Asia and Oceania Perinatal Societies, the International Society of Perinatal Obstet. 2013;26(2):137–42. 10.3109/14767058.2012.733744 23013166

[24] Wagstrom EA, Yoon KJ, Zimmerman JJ. Immune components in porcine mammary secretions. Viral Immunol. 2000;13(3):383–97. 10.1089/08828240050144699 11016601

[25] Meganck V, Opsomer G, Piepers S, Cox E, Goddeeris BM. Maternal colostral leukocytes appear to enhance cell-mediated recall response, but inhibit humoral recall response in prime-boost vaccinated calves. Journal of reproductive immunology. 2016;113:68–75. 10.1016/j.jri.2015.11.004 26796988

[26] Gerjets I, Kemper N. Coliform mastitis in sows: A review. J Swine Health Prod. 2009;17(2):97–105.

[27] Persson A, Pedersen AE, Goransson L, Kuhl W. A Long-Term Study on the Health-Status and Performance of Sows on Different Feed Allowances during Late Pregnancy.1. Clinical Observations, with Special Reference to Agalactia Post Partum. Acta Vet Scand. 1989;30(1):9–17. 2782237 10.1186/BF03548063 PMC8142194

[28] Dawson H, Lunney J. Porcine cluster of differentiation (CD) markers 2018 update. Res Vet Sci. 2018;118. 10.1016/j.rvsc.2018.02.007 29518710

[29] Foss DL, Murtaugh MP. Mucosal immunogenicity and adjuvanticity of cholera toxin in swine. Vaccine. 1999;17(7–8):788–801. 10.1016/s0264-410x(98)00263-1 10067684

[30] Demeckova V, Kelly D, Coutts AG, Brooks PH, Campbell A. The effect of fermented liquid feeding on the faecal microbiology and colostrum quality of farrowing sows. International journal of food microbiology. 2002;79(1–2):85–97. 10.1016/s0168-1605(02)00182-4 12382688

[31] SAS. System for Microsoft Windows. In: Cary, editor. Release 94. NC, USA: SAS INSTITUTE INC.; 2002–2012.

[32] Salmon H, Delouis C. Kinetics of Lymphocyte Sub-Populations and Plasma-Cells in the Mammary-Gland of Primiparous Sows in Relation to Gestation and Lactation. Ann Rech Vet. 1982;13(1):41–9. 7165262

[33] Meganck V, Goddeeris BM, De Campeneere S, Hostens M, Van Eetvelde M, Piepers S, et al. Effect of beta-hydroxybutyric acid, parity, and body condition score on phenotype and proliferative capacity of colostral mononuclear leukocytes of high-yielding dairy cows. J Dairy Sci. 2015;98(10):6782–91. 10.3168/jds.2014-8780 26233460

[34] Ohtsuka H, Terasawa S, Watanabe C, Kohiruimaki M, Mukai M, Ando T, et al. Effect of parity on lymphocytes in peripheral blood and colostrum of healthy Holstein dairy cows. Can J Vet Res. 2010;74(2):130–5. 20592843 PMC2851723

[35] Islam SK, Ahmed L, Khan MN, Huque S, Begum A, Yunus AB. Immune components (IgA, IgM, IgG, immune cells) of colostrum of Bangladeshi mothers. Pediatrics international: official journal of the Japan Pediatric Society. 2006;48(6):543–8. 10.1111/j.1442-200X.2006.02291.x 17168971

[36] Park YH, Fox LK, Hamilton MJ, Davis WC. Bovine mononuclear leukocyte subpopulations in peripheral blood and mammary gland secretions during lactation. J Dairy Sci. 1992;75(4):998–1006. 10.3168/jds.S0022-0302(92)77842-4 1578038

[37] Yang TJ, Ayoub IA, Rewinski MJ. Lactation stage-dependent changes of lymphocyte subpopulations in mammary secretions: inversion of CD4+/CD8+ T cell ratios at parturition. American journal of reproductive immunology. 1997;37(5):378–83. 10.1111/j.1600-0897.1997.tb00247.x 9196796

[38] Okutani M, Tsukahara T, Kato Y, Fukuta K, Inoue R. Gene expression profiles of CD4/CD8 double-positive T cells in porcine peripheral blood. Animal science journal = Nihon chikusan Gakkaiho. 2018;89(7):979–87. 10.1111/asj.13021 29740910

[39] Ogawa S, Okutani M, Tsukahara T, Nakanishi N, Kato Y, Fukuta K, et al. Comparison of gene expression profiles of T cells in porcine colostrum and peripheral blood. American journal of veterinary research. 2016;77(9):961–8. 10.2460/ajvr.77.9.961 27580107

[40] Rodriguez-Gomez IM, Talker SC, Kaser T, Stadler M, Hammer SE, Saalmuller A, et al. Expression of T-bet, Eomesodermin and GATA-3 in porcine alphabeta T cells. Dev Comp Immunol. 2016;60:115–26. 10.1016/j.dci.2016.02.022 26920461

[41] Zuckermann FA, Gaskins HR. Distribution of porcine CD4/CD8 double-positive T lymphocytes in mucosa-associated lymphoid tissues. Immunology. 1996;87(3):493–9. 8778039 PMC1384122

[42] Zuckermann FA, Husmann RJ. Functional and phenotypic analysis of porcine peripheral blood CD4/CD8 double-positive T cells. Immunology. 1996;87(3):500–12. 8778040 PMC1384123

[43] Talker SC, Kaser T, Reutner K, Sedlak C, Mair KH, Koinig H, et al. Phenotypic maturation of porcine NK- and T-cell subsets. Dev Comp Immunol. 2013;40(1):51–68. 10.1016/j.dci.2013.01.003 23352625

[44] Borghetti P, De Angelis E, Saleri R, Cavalli V, Cacchioli A, Corradi A, et al. Peripheral T lymphocyte changes in neonatal piglets: Relationship with growth hormone (GH), prolactin (PRL) and cortisol changes. Vet Immunol Immunopathol. 2006;110(1–2):17–25. 10.1016/j.vetimm.2005.09.001 16213031

[45] Xanthou M. Immune protection of human milk. Biol Neonate. 1998;74(2):121–33. 10.1159/000014018 9691154

[46] Stepanova K, Sinkora M. The expression of CD25, CD11b, SWC1, SWC7, MHC-II, and family of CD45 molecules can be used to characterize different stages of gamma delta T lymphocytes in pigs. Dev Comp Immunol. 2012;36(4):728–40. 10.1016/j.dci.2011.11.003 22100879

[47] Nguyen TV, Yuan LJ, Azevedo MSP, Jeong KI, Gonzalez AM, Saif LJ. Transfer of maternal cytokines to suckling piglets: In vivo and in vitro models with implications for immunomodulation of neonatal immunity. Vet Immunol Immunop. 2007;117(3–4):236–48. 10.1016/j.vetimm.2007.02.013 17403542 PMC4094377

[48] Laskowska E, Jarosz L, Gradzki Z. Effect of Multi-Microbial Probiotic Formulation Bokashi on Pro- and Anti-Inflammatory Cytokines Profile in the Serum, Colostrum and Milk of Sows, and in a Culture of Polymorphonuclear Cells Isolated from Colostrum. Probiotics and antimicrobial proteins. 2019;11(1):220–32. 10.1007/s12602-017-9380-9 29305686 PMC6449489

[49] Wilson SM, Wilkie BN. B-1 and B-2B-cells in the pig cannot be differentiated by expression of CD5. Vet Immunol Immunop. 2007;115(1–2):10–6. 10.1016/j.vetimm.2006.10.009 17098293

[50] Berland R, Wortis HH. Origins and functions of B-1 cells with notes on the role of CD5. Annual review of immunology. 2002;20:253–300. 10.1146/annurev.immunol.20.100301.064833 11861604

[51] Moreno S, Alvarez B, Martinez P, Uenishi H, Revilla C, Ezquerra A, et al. Analysis of chemokine receptor CCR7 expression on porcine blood T lymphocytes using a CCL19-Fc fusion protein. Dev Comp Immunol. 2013;39(3):207–13. 10.1016/j.dci.2012.11.010 23219903

[52] Cabrera RA, Lin X, Campbell JM, Moeser AJ, Odle J. Influence of birth order, birth weight, colostrum and serum immunoglobulin G on neonatal piglet survival. Journal of animal science and biotechnology. 2012;3(1):42. 10.1186/2049-1891-3-42 23259926 PMC3541264

[53] Quesnel H, Farmer C, Devillers N. Colostrum intake: Influence on piglet performance and factors of variation. Livest Sci. 2012;146(2–3):105–14.

[54] Devillers N, Farmer C, Le Dividich J, Prunier A. Variability of colostrum yield and colostrum intake in pigs. Animal. 2007;1(7):1033–41. 10.1017/S175173110700016X 22444806

[55] Bland IM, Rooke JA, Bland VC, Sinclair AG, Edwards SA. Appearance of immunoglobulin G in the plasma of piglets following intake of colostrum, with or without a delay in sucking. Anim Sci. 2003;77:277–86.

[56] Hurley WL, Theil PK. Perspectives on immunoglobulins in colostrum and milk. Nutrients. 2011;3(4):442–74. 10.3390/nu3040442 22254105 PMC3257684

[57] Carney-Hinkle EE, Tran H, Bundy JW, Moreno R, Miller PS, Burkey TE. Effect of dam parity on litter performance, transfer of passive immunity, and progeny microbial ecology. J Anim Sci. 2013;91(6):2885–93. 10.2527/jas.2011-4874 23482585

[58] Lipkin M. Growth and Development of Gastrointestinal Cells. Annu Rev Physiol. 1985;47:175–97. 10.1146/annurev.ph.47.030185.001135 3888073

[59] Donovan SM, Zijlstra RT, Odle J. Use of the piglet to study the role of growth factors in neonatal intestinal development. Endocrine regulations. 1994;28(4):153–62. 7711292

[60] Jaeger LA, Lamar CH, Bottoms GD, Cline TR. Growth-stimulating substances in porcine milk. American journal of veterinary research. 1987;48(10):1531–3. 3314608

[61] Xu RJ, Mellor DJ, Birtles MJ, Breier BH, Gluckman PD. Effects of oral IGF-I or IGF-II on digestive organ growth in newborn piglets. Biol Neonate. 1994;66(5):280–7. 10.1159/000244118 7533009

[62] Chen T, Xi QY, Ye RS, Cheng X, Qi QE, Wang SB, et al. Exploration of microRNAs in porcine milk exosomes. BMC genomics. 2014;15:100. 10.1186/1471-2164-15-100 24499489 PMC4008308

[63] Salcedo J, Frese SA, Mills DA, Barile D. Characterization of porcine milk oligosaccharides during early lactation and their relation to the fecal microbiome. J Dairy Sci. 2016;99(10):7733–43. 10.3168/jds.2016-10966 27522435 PMC5557353

[64] Tao N, Ochonicky KL, German JB, Donovan SM, Lebrilla CB. Structural Determination and Daily Variations of Porcine Milk Oligosaccharides. J Agr Food Chem. 2010;58(8):4653–9. 10.1021/jf100398u 20369835 PMC2882034

[65] Picone G, Zappaterra M, Luise D, Trimigno A, Capozzi F, Motta V, et al. Metabolomics characterization of colostrum in three sow breeds and its influences on piglets’ survival and litter growth rates. Journal of animal science and biotechnology. 2018;9:23. 10.1186/s40104-018-0237-1 29527304 PMC5840723

[66] Martin R, Delgado S, Maldonado A, Jimenez E, Olivares M, Fernandez L, et al. Isolation of lactobacilli from sow milk and evaluation of their probiotic potential. J Dairy Res. 2009;76(4):418–25. 10.1017/S0022029909990124 19640313

[67] Ferrari CV, Sbardella PE, Bernardi ML, Coutinho ML, Vaz IS Jr., Wentz I, et al. Effect of birth weight and colostrum intake on mortality and performance of piglets after cross-fostering in sows of different parities. Preventive veterinary medicine. 2014;114(3–4):259–66. 10.1016/j.prevetmed.2014.02.013 24674020

