# Correction: Distribution difference of colostrum-derived B and T cells subsets in gilts and sows

**DOI:** 10.1371/journal.pone.0305647

**Published:** 2024-06-12

**Authors:** The PLOS ONE Staff

There is an error in Table 1. For lines 7, 8, 11, 12, 13, 16, 18, 19, 20, 21, 22, 23 and 24, the value in the column “Working Dilution” is not “10-Jan.” The correct value “1/10.” The publisher apologizes for the error.

**Table 1:** Antibodies used in Flow Cytometry.

Antibody	Species	Clone	Fluorochrome	Labeling strategy	Isotype	Concentration (mg/ml)	Working dilution
7-AAD	NA	NA	7-AAD	Dye	NA	0.1	1/400
granulocyte	mouse anti-pig	6D10	FITC	Directly conjugated	IgG2a	0.1	Neat
CD45RA	mouse anti-pig	MIL13	FITC	Directly conjugated	IgG1	0.1	Neat
CD4alpha	mouse anti-pig	MIL17	FITC	Directly conjugated	IgG2b	0.1	Neat
IgM	goat anti-pig	AAI48	FITC	Directly conjugated	IgM	1.0	1/100
CD5	mouse anti-pig	1H6/8	FITC	Directly conjugated	IgG2a	0.1	Neat
CD14	mouse anti-pig	MIL2	FITC	Directly conjugated	IgG2b	0.1	1/10
CD79a	Mouse anti-human	HM57	RPE	Directly conjugated	IgG1	Not determined	1/10
CD4alpha	mouse anti-pig	MIL17	RPE	Directly conjugated	IgG2b	Not determined	1/100
CD8alpha	mouse anti-pig	MIL12	RPE	Directly conjugated	IgG2a	Not determined	1/100
CD45RA	mouse anti-pig	MIL13	RPE	Directly conjugated	IgG1	Not determined	1/10
CD16	mouse anti-pig	G7	RPE	Directly conjugated	IgG1	Not determined	1/10
CD3	mouse anti-pig	PPT3	RPE-Cy7^a^	Secondary antibody^a^	IgG1	0.1	1/10
SWC7 or WC4	mouse anti-bovine	CC55	RPE-Cy7^a^	Secondary antibody^a^	IgG1	0.1	Neat
macrophages	mouse anti-pig	BA4D5	APC^b^	Secondary antibody^b^	IgG2b	0.1	Neat
SWC2 or CD27	mouse anti-pig	B30C7	APC	Directly conjugated	IgG1	Not determined	1/10
CD335	mouse anti-pig	VIV-KM1	APC	Directly conjugated	IgG1	Not determined	1/100
IgG1 isotype control	mouse	NA	FITC	Directly conjugated	IgG1	0.1	1/10
IgG2a isotype control	mouse	NA	FITC	Directly conjugated	IgG2a	0.1	1/10
IgG2b isotype control	mouse	NA	FITC	Directly conjugated	IgG2b	0.1	1/10
IgG1 isotype control	mouse	NA	RPE	Directly conjugated	IgG1	Not determined	1/10
IgG2a isotype control	mouse	NA	RPE	Directly conjugated	IgG2a	Not determined	1/10
IgG2b isotype control	mouse	NA	RPE	Directly conjugated	IgG2b	Not determined	1/10
IgG1 isotype control	mouse	NA	APC	Directly conjugated	IgG1	Not determined	1/10
IgG1 isotype control	mouse anti-pig	NA	RPE-CY7^a^	Secondary antibody^a^	IgG1	0.1	Neat

*a* MAb dilution following PE-Cy7 labeling (Serotec).
